# Targeting the Tumor Microenvironment: Focus on Angiogenesis

**DOI:** 10.1155/2012/281261

**Published:** 2011-08-24

**Authors:** Fengjuan Fan, Alexander Schimming, Dirk Jaeger, Klaus Podar

**Affiliations:** Medical Oncology, National Center for Tumor Diseases (NCT), University of Heidelberg and German Cancer Research Center (DKFZ), Im Neuenheimer Feld 460, 69120 Heidelberg, Germany

## Abstract

Tumorigenesis is a complex multistep process involving not only genetic and epigenetic changes in the tumor cell but also selective supportive conditions of the deregulated tumor microenvironment. One key compartment of the microenvironment is the vascular niche. The role of angiogenesis in solid tumors but also in hematologic malignancies is now well established. Research on angiogenesis in general, and vascular endothelial growth factor in particular, is a major focus in biomedicine and has led to the clinical approval of several antiangiogenic agents including thalidomide, bevacizumab, sorafenib, sunitinib, pazopanib, temesirolimus, and everolimus. Indeed, antiangiogenic agents have significantly changed treatment strategies in solid tumors (colorectal cancer, renal cell carcinoma, and breast cancer) and multiple myeloma. Here we illustrate important aspects in the interrelationship between tumor cells and the microenvironment leading to tumor progression, with focus on angiogenesis, and summarize derived targeted therapies.

## 1. Introduction

Cancer research in both solid and hematologic malignancies until recently predominantly focused on the identification of genetic changes which are intimately associated with the induction and progression of tumors and metastasis [1]. A variety of multistep tumor models with accumulating somatic mutations has been proposed [2], most prominently the multistep colon cancer model of Dr. Vogelstein's group [3, 4]. In addition to focal genetic lesions (point mutations), chromosomal aberrations (e.g., aneuploidy, translocations, chromosomal deletions) as well as epigenetic alterations (e.g., DNA methylation, histone deacetylation, nucleosome remodeling, and RNA-associated silencing) induce deregulated expression of oncogenes and suppressor genes thereby leading to tumor cell proliferation, transformation and invasion [5, 6]. Recent studies add yet another facet to the complex multistep model of tumorigenesis by demonstrating that tumor cells carrying genomic and epigenomic abnormalities also trigger changes in their microenvironment. In turn, these changes enable the formation of a selective supportive “tumor microenvironment” [7, 8].

The cellular tumor microenvironment that is, the bone marrow microenviroment is composed of *nonhematopoietic cells* including endothelial cells (ECs); cancer-associated fibroblasts (CAFs); and cells involved in bone homeostasis including chondroblasts, osteoclasts, and osteoblasts; and *hematopoietic cells* including immune cells (including natural killer cells (NK) cells, tumor-associated macrophages (TAMs), T lymphocytes, monocytes); erythrocytes; megakaryocytes and platelets; stem cells; progenitor and precursor cells; and circulating endothelial precursors (CEPs). The noncellular microenvironment is composed of the *extracellular matrix* (*ECM) *proteins including fibronectin, laminin, collagen, osteopontin, proteoglycans, and glycosaminoglycans—and the *liquid milieu* (cytokines and growth factors, proteases) (Table 1). Tumor cell-induced disruption of the microenvironment homeostasis between the highly organized cellular and extracellular compartments support sustained proliferative signaling, evade growth suppressors, resist cell death, enable replicative immortality, activate invasion and metastasis, reprogram energy metabolism, evade immune destruction, and induce drug resistance and angiogenesis. Based on our enhanced understanding of the functional importance of the tumor microenvironment and tumor angiogenesis, in particular, new molecular targets have been identified. 

This paper aims to illustrate important aspects in the interrelationship between tumor cells and the tumor microenvironment, tumor angiogenesis in particular, in tumor progression. Four tumor entities, in which antiangiogenic agents have already significantly changed treatment strategies, are taken as examples: colorectal cancer (CRC), renal cell carcinoma (RCC), and breast cancer (BC), as well as multiple myeloma (MM).

## 2. Tumor Angiogenesis

Research on tumor angiogenesis is a major focus in biomedicine. Historically, Dr. Virchow was the first to identify a huge number of blood vessels in tumors in 1863 [13]. Few decades later in 1907, Goldman was the first to describe tumor vascularization in carcinomas of the stomach, the liver, and other organs [14]. In 1913, Murphy reported about the angiogenic response induced by Jensen rat sarcoma cells in the chick chorioallantoic membrane (CAM) [15]. The term “angiogenesis” was first used in 1935 and described the formation of new blood vessels in the placenta [16] and four years later in wound healing and tumor growth [17]. However, it was not until 1971 when Folkman hypothesized that inhibition of angiogenesis may be a potential way to inhibit cancer progression [18]. Subsequently, independent studies by Senger and Dvorak, Ferrara and Henzel, as well as Connolly and colleagues led to the purification, identification and cloning of vascular endothelial growth factor (VEGF), the key proangiogenic factor [19–23]. Since then, our knowledge of molecular mechanisms to tumor angiogenesis continually increased leading to the discovery of promising antiangiogenic therapies for tumor patients [24, 25]. Specifically, the impact of the tumor microenvironment, and tumor angiogenesis in particular, has been studied in greater detail in three types of solid cancer (CRC, RCC, BC)—and MM. These diseases serve as paradigm diseases for ongoing studies also in other tumor entities. 

During tumorigenesis, the appropriate balance between proangiogenic and antiangiogenic molecules which arise from cancer cells and stromal cells in response to direct cell-cell, cell-ECM binding as well as to autocrine and paracrine growth factor stimulation, is lost [26, 27]. The “angiogenic switch”, a rapid increase of blood vessel formation to support tumor growth, is triggered by (1) oncogene-mediated tumor expression of angiogenic proteins including VEGF, fibroblast growth factor (FGF), platelet derived growth factor (PDGF), endothelial growth factor (EGF), lysophosphatic acid (LPA), and angiopoietin (Ang), (2) metabolic and/or mechanical stress, (3) genetic mutations, (4) the immune response, and maybe most prominently (5) hypoxia. Tumor-angiogenesis therefore depends on tumor type, site, growth, and stage of disease and contributes to tumor growth, invasion, and metastasis. 

The main mechanism of tumor angiogenesis is endothelial sprouting which crucially depends on VEGF upregulation and the interaction between ECs, pericytes, stroma cells as well as their association with the ECM [28, 29]. Specifically, VEGF and angiopoietin activate matrix degrading enzymes including the plasminogen activator (PA) and matrix metalloproteinases (MMPs) to loosen the matrix and favor EC migration [30]. VEGF and angiopoietin-2 (Ang-2)/type I tyrosine kinase receptor 2 (TIE 2) system then induce the detachment of pericytes and thereby increase vessel porosity. Plasma proteins are exuded and provide a gradient for EC migration [31–34]. Mechanistically, vessel sprouting is mediated by specialized ECs: tip cells lead the new sprout; stalk cells trail behind the pioneering tip cell, proliferate to form an elongating, stalk and create a lumen; and endothelial nonproliferating phalanx cells sense and regulate perfusion in the persistent sprout. Functionally, VEGF induces both NOTCH 1-mediated proliferation in stalk cells as well as directed migration of delta-like 4 (DLL 4)-expressing tip cells towards the sources of angiogenic factors. Endothelial cell-derived factor epidermal growth factor-like domain multiple 7 (Egfl 7) and components of the ECM then regulate vascular lumen formation [35]. Finally, PDGF produced by ECs then recruits pericytes, which surround and stabilize new vessels.

Besides sprouting, the formation of the endothelial lining of tumor vessels is promoted by cooption of neighboring preexisting vessels [36], intussusception (insertion of connective tissue columns into vessel lumen), glomeruloid angiogenesis, as well as VEGF-induced recruitment of highly proliferative circulating endothelial cells (CECs) and endothelial progenitor cells (EPCs) from the BM, hematopoietic stem cells (HSCs), progenitor cells, monocytes, and macrophages [37]. In addition, tumor cells themselves act as ECs to form functional avascular blood conduits or mosaic blood vessels [38–42]. 

Oxygen tension is the key regulator of VEGF expression, predominantly via the hypoxia-inducible factor (HIF)/von Hippel-Lindau tumor suppressor gene (VHL) pathway. Under normoxic conditions, prolyl hydroxylase domain (PHD) proteins hydroxylate prolyl residues on HIF, which are recognized by VHL, polyubiquitinated, and undergo proteasomal degradation. Tumor growth is often accompanied by a decrease in oxygen tension due to insufficient vascularization [43]. In turn, the process of tumor angiogenesis gets initiated and blood vessels supply nutrients and oxygen for the tumors that reach a hypoxic and necrotic area [43]. Under hypoxic conditions, PHD proteins are inactive, and nonhydroxylated HIF accumulates, translocates to the nucleus and binds to hypoxia-response elements (HRE) thereby initiating transcription of various genes that play a central part in angiogenesis. Genes induced by HIF include VEGF, PDGF, transforming growth factor-*β* (TGF-*β*), TGF*α*, epidermal growth factor receptor (EGFR), insulin-like growth factor 2 (IGF2), MMP1, stromal cell-derived factor 1 (SDF1), glucose transporter 1 (involved in glucose metabolism), as well as carbonic anhydrase 9 (CAIX), and activin B [44–47]. Factor inhibiting HIF (FIH) modulates interaction of HIF with the coactivators CBP/p300 [48]. HIF is also regulated by oxygen-independent pathways via growth-factor receptors or other signaling molecules. Specifically, growth factors, signaling molecules, and loss of function mutations of molecules such as VHL, p53, and PTEN, trigger HIF-1*α* synthesis. HIF expression is also controlled by specific microRNAs. A recent study identified a unique microRNA in hypoxic endothelia cells, miR424, that promotes HIF-1 stabilization and angiogenesis [49, 50]. Importantly, besides being a key regulator of angiogenesis, HIF activity is required for tumor cell survival and proliferation, migration, invasion, pH regulation, metabolism, drug and radiation resistance, immune evasion, and genetic stability [51, 52].

## 3. Colorectal Cancer

Major improvements in the therapy of CRC have been made during the last decades. These improvements are based on our increased knowledge of the role of the tumor microenvironment, and angiogenesis in particular, in CRC tumorigenesis. In the late 1980s, Dr. Vogelstein postulated a paradigm of multistep carcinogenesis in CRC involving a progressive series of specific and well-defined genetic alterations in tumor suppressor genes (APC, p53, or DCC) and in oncogenes (K-Ras), which render normal mucosa to carcinoma [53, 54]. Besides inducing tumor cell proliferation, survival, migration, and drug resistance, these alterations trigger changes in the tumor microenvironment, tumor angiogenesis in particular, *via* upregulation of VEGF as well as deregulation of other molecules including EGFR and COX2. Increased levels of VEGF and EGFR expression have been found in patients with localized as well as metastatic CRC [55–60]. Based on successful clinical phase III trials both VEGF inhibitors (e.g., bevacizumab) as well as EGFR inhibitors (e.g., cetuximab, panitumumab) have been approved and incorporated into novel treatment regimens of progressed CRC. 

Metabolic products of cyclooxygenase 2 (COX2), prostaglandins in particular, contribute to neovascularisation and support vasculature-dependent growth of CRC, invasion, and metastasis [31, 61, 62]. COX2 is upregulated in approximately 50% of adenomas and 85% of adenocarcinomas [63, 64] and associated with worse survival among CRC patients [65]. Genetic deletion of COX2 dramatically reduces intestinal polyp formation supporting a key function of COX2 in CRC tumorigenesis [66]. Functionally, COX2 triggers secretion of MMP2 and MMP9 and enhances the expression of proangiogenic growth factors including VEGF and bFGF. It therefore contributes to the dissolution of the collagen matrix, EC migration, and formation of tubular networks [67–70]. COX2 inhibitors suppress VEGF and bFGF expression and thereby block angiogenesis [71–73]. Indeed, both aspirin and nonaspirin-NSAIDs given daily reduce the incidence of CRC significantly [74, 75].

Another potential therapeutic target is endoglin, a membrane-steady TGF*β* coreceptor regulating tumorangiogenesis in CRC [76, 77]. High levels of soluble Endoglin have been found in CRC and BC patients [78] where it contributes to EC dysfunction [79, 80]. However, exact mechanism of soluble endoglin on tumor angiogenesis remain to be identified. 

In summary, inhibitors of growth factors contributing to tumor angiogenesis such as VEGF, EGF, and also COX2 have already been incorporated into novel treatment regimens and maintenance therapies in CRC. Promising future therapeutic targets include endoglin.

## 4. Renal Cell Carcinoma

Renal cell carcinoma/hypernephrom accounts for 2-3% of all cancer cases in adults. It is the seventh most common cancer in men and the ninth most common in women [81]. While localized RCC has a 5-year survival rate of 60–70%, metastatic RCC is the most lethal of all urological cancers [82]. Resistant to chemotherapy [81], only immunotherapy with IL-2 and interferon *α* (IFN *α*) has been utilized for systemic RCC therapy until most recently [83]. The introduction of antiangiogenic agents has dramatically improved treatment options in metastatic RCC. Indeed, an unprecedented six antiangiogenic agents have been approved for RCC treatment during the last 5 years including sunitinib, temsirolimus, everolimus, pazopanib, bevacizumab, and sorafenib. These agents improve progression-free survival. However, improvements of overall survival have not been demonstrated yet.

The evaluation of antiangiogenic agents for treatment of RCC has been triggered by the finding that RCC is a highly vascular tumor and that increased microvessel density (MVD) correlates with increased risk of metastasis, recurrence and adverse prognosis. High expression of VEGF and other angiogenic factors are predominantly triggered by the inactivation of the VHL tumor suppressor gene due to the loss of 3p [84–88]. Consequently, HIF is not degraded even under normoxic conditions [85]. Furthermore, VHL has many functions that are independent of HIF [89]. For example, inactivated VHL cannot interact with fibronectin and hydroxylated collagen IV. It thereby leads to impaired ECM organization invasion and angiogenesis in RCC [90, 91].

Besides VHL/HIF signaling, other signaling pathways may also participate in the regulation of secreted angiogenic factors in RCC. For example, in VHL-defective RCC cells, oncoprotein HDM2 not only affects constitutively expressed HIF*α*, but also directly regulates protein levels of HIF angiogenic targets (e.g., VEGF, PA inhibitor-1 (PAI-1), and endothelin-1 (ET-1)) [92].

RCC is one of the most immunogenic tumors [93]. Importantly, besides its effects on angiogenesis VEGF modulates immune tolerance in the tumor microenvironment by attenuating dendritic cell differentiation [94], and increasing secretion of immunosuppressive cytokines [95]. Anti-RCC activity of VEGF-inhibitors may therefore, at least in part, also be mediated *via* modulation of the antitumor immunity.

## 5. Breast Cancer

In 2010, BC was the cancer with the most new cases (207,090 women) of females in the USA and forth highest death rate (39,840 women) [96]. As in CRC and RCC, VEGF expression is also upregulated in BC. Moreover, angiogenesis represents a major independent prognostic factor in BC [97]. VEGF production and secretion within the BC microenvironment is triggered by a number of stimuli including growth factors, cytokines, hormones, loss of p53 function, RAS and SRC mutations, hypoxia as well as overexpression of HER2 (HER2/neu, ErbB2) [98–100]. Moreover, high levels of MMP-9 are produced and secreted by BC cells [101] and release sequestered VEGF from the adjacent ECM [102]. Importantly, VEGF levels are higher in premenopausal patients than in postmenopausal patients indicating that steroid hormones increase VEGF expression [103]. Indeed, upregulation of VEGF in tumor cell lines is triggered by the interaction of the ER*α*/estradiol-complex with an imperfect estrogen response element located 1.5 kb upstream of the VEGF transcription start site [104, 105]. 

HER2 is a member of the EGFR family encoded by the ERB2 gene. In human BC, the HER2 gene is amplified in 20–30% of all BC [106, 107]. Phosphorylation of the tyrosine kinase domain results in tumor cell and EC proliferation and survival *via* PI3K- and Ras/MEK/MAPK-signaling pathways [57, 108, 109]. In addition to phosphorylation, cleavage of the extracellular domain of HER2 generates an intracellular domain (p95) which activates these signaling pathways. 

Another regulator of angiogenesis in BC is osteoprotegerin (OPG), a glycoprotein belonging to the TNF receptor (TNFR) superfamily whose production is triggered by direct cell-EC contact [110]. High levels of OPG are present in tumor ECs and correlate with tumor grade in BC [111]. 

Similarly, the transcription factor HOXB9 is overexpressed in 42% of patients with BC. It induces production of TGF-*β*, ErbB ligands, and several angiogenic factors (VEGF, bFGF, IL-8, and ANGPTL-2) thereby resulting in the induction of mesenchymal cell fate, invasion, as well as angiogenesis [112].

Finally, *fes* proto-oncogene (also known as *fps*) which encodes a Src homology 2 (SH2) domain-containing cytoplasmic PTK mediates tumor angiogenesis and metastasis [113]. Indeed the tumor microenvironment in Fes-deficient mice showed reduced vascularity and fewer tumor-associated macrophages indicating a therapeutic role for fes-inhibition [114].

In addition to bevacizumab, a variety of additional antiangiogenic agents is under clinical investigation for treatment of BC in the palliative as well as in the adjuvant setting. Importantly, also the anti-BC activity of tamoxifen is, at least in part, due to its antiangiogenic effect [115–117].

## 6. Multiple Myeloma

MM is a B-cell neoplasm characterized by excess clonal proliferation of malignant plasma cells in the bone marrow, elevated serum and urine monoclonal protein, osteolytic bone lesions, renal disease, and immunodeficiency. MM is the second most frequent malignancy of the blood in the USA. It causes about 1% of neoplastic diseases and 13% of hematological malignancies [118, 119]. The development of MM involves both early and late genetic changes in the tumor cell as well as selective supportive conditions by the bone marrow (BM) microenvironment, BM angiogenesis in particular [120]. It is suggested that MGUS and nonactive MM in which the tumor growth is arrested are “avascular phases” of plasma cell tumors, while the active MM is the “vascular phase”, which is associated with clonal expansion and epigenetic modifications of the microenvironment as well as the “angiogenic switch” [121, 122]. Importantly, these findings correlate with disease progression and poor prognosis. Moreover, BM MVD at the time of initial diagnosis is an important prognostic factor for median overall survival (OS) and median progression-free survival (PFS) in patients undergoing autologous transplantation as frontline therapy for MM [123].

VEGF within the MM BM microenvironment induces growth, survival as well as migration of MM cells in an autocrine manner via VEGFR-1 and triggers angiogenesis via VEGF-2 in ECs [122–127]. Recent studies suggest the existence of MM-specific ECs (MMECs) which produce growth and invasive factors for plasma cells, including VEGF, FGF-2, MMP-2 as well as MMP-9. Compared to healthy human umbilical vein EC (HUVEC), MMECs secrete higher amounts of the CXC chemokines (e.g., IL8, SDF1-*α*, MCP-1), which act in a paracrine manner to mediate plasma cell proliferation and chemotaxis [120–123, 126, 128, 129]. In turn, MM cells and stromal cells prolong survival of ECs both by increased secretion of EC survival factors, such as VEGF, and by decreased secretion of antiangiogenic factors [123, 130, 131]. 

Based on the enhanced understanding of the functional importance of the MM BM microenvironment and its interrelation with the MM cell resulting in homing, seeding, proliferation and survival, new molecular targets have been identified and derived treatment regimens in MM have already changed fundamentally during recent years. The anti-MM activity of thalidomide, bortezomib, and lenalidomide is mediated, at least in part, also via antiangiogenic effects [132]. For the treatment of MM, additional antiangiogenic therapies are therefore being evaluated in combination with conventional or novel anti-MM therapies [12, 127].

## 7. Inhibitors of Angiogenesis (Table 2)

### 7.1. Thalidomide and the IMiDs (Lenalidomide/Revlimid, Pomalidomide/Actimid)

In 1994, D'Amato et al. studied the mechanism of thalidomide's teratogenicity and found that thalidomide (Celgene) is a potential inhibitor of angiogenesis [133]. Based on this finding and the discovery that bone marrow MVD plays a key role in MM pathogenesis, thalidomide was used empirically to treat patients with refractory relapsed MM in the late 90s. Remarkable clinical responses rendered thalidomide to be the first antiangiogenic agent for cancer treatment [134]. Currently, thalidomide is not only used in patients with refractory/relapsed but also with newly diagnosed MM. 

Subsequently, a series of thalidomide-derived immunomodulatory drugs (IMiDs) including lenalidomide (Revlimid) and pomalidomide (Actimid) have been developed [135]. A phase I dose-escalation trial using lenalidomide in patients with relapsed and refractory MM demonstrated either response or stabilization of disease in 79% cases [136]. Two clinical phase II trials confirmed these data and achieved complete responses with favorable side effect profiles; two clinical phase III trials comparing lenalidomide to dexamethasone/lenalidomide treatment of relapsed MM provided the basic for its FDA approval in 2006. In the relapsed/refractory setting an overall response of 30% was achieved by the new IMID pomalidomide, alone or in combination with dexamethasone. More than 100 clinical studies with thalidomide or lenalidomide combined with other agents are currently recruiting or ongoing. 

Adverse side effects of thalidomide and the IMiDs include polyneuropathy, fatigue, skin rash, and venous thromboembolism (VTE), or blood clots, which could lead to stroke or myocardial infarction. Both thalidomide and the IMiDs overcome the growth and survival advantage conferred by the BM milieu, at least in part by downregulating VEGF [137, 138], and inhibition of proliferation and capillarogenesis of MMECs [128].

### 7.2. Bevacizumab (Avastin)

Bevacizumab (Genetech) [139] binds biologically active forms of VEGF and prevents its interaction with VEGF receptors (VEGFR-1 and VEGFR-2), thereby inhibiting endothelial cell proliferation and angiogenesis. In preclinical studies bevacizumab reduced microvascular growth and inhibited metastasis of colon growth in nude mice [140–142]. 

When tested in patients with metastatic CRC bevacizumab in combination with conventional chemotherapy demonstrated significant survival benefits. Based on this finding, the US FDA approved bevacizumab in February 2004, followed by the EMEA approval in January 2005, as first-line treatment of metastatic CRC in combination with 5-fluorouracil-(FU-) based chemotherapy regimens. In 2006, bevacizumab in combination with 5-FU was also approved for second-line treatment of CRC. In contrast, the use of bevacizumab in the adjuvant setting cannot be recommended [143–145]. Bevacizumab is therefore the first VEGF-targeting agent approved both by the US FDA as well as the EMEA for cancer treatment [146]. 

Since its initial approval as first-line treatment in metastatic CRC in 2004, bevacizumab has been approved for use in combination with other chemotherapeutics in four other tumor types: in 2009 (US) and 2007 (EU) for advanced RCC, in 2008 for metastatic HER2-negative BC, in 2009 for glioblastoma, and in 2004 for non-small cell lung cancer (NSCLC) [147–150].

Specifically, the E2100 study was the first Phase III study using bevacizumab in metastatic BC as first-line treatment. Bevacizumab was investigated in combination with and without paclitaxel. In combination with bevacizumab, progression-free survival was doubled (5.8 months to 11.3 months). The overall response rate increased from 22 to 50%. Because of this study, bevacizumab was approved for metastatic BC [151]. But as the overall survival did not show any benefit, Fojo and Wilkerson [152] believe that the E2100 trial overestimated the benefit of bevacizumab and that further studies need to target the VEGF polymorphism of VEGF in order to identify the patients that derive true benefit from bevacizumab [153]. Based on two double-blind studies (AVADO and RIBBON-1) showing high toxicity without significant improvements of progression-free survival [154–156], the use of bevacizumab as first-line therapy in progressed Her2-negative BC has been removed by the US FDA in 2010. In a meta-analysis, Ranpura et al. report that addition of bevacizumab to systemic antineoplastic therapy is associated with a significantly increased risk (relative risk of 1.46; incidence, 2.5% versus 1.7%) of fatal adverse events (FAEs), in BC patients [157, 158]. However, clinical studies evaluating bevacizumab in combination with conventional therapies both in Her2-negative and also Her2-positive patients are ongoing. It may be possible to focus bevacizumab treatment in patients most likely to benefit, and avoid treatment of patients unlikely to benefit or more likely to experience toxic effects [157]. 

Although generally well tolerated, side effects of bevacizumab treatment include minor (hypertension, proteinuria, nosebleed, upper respiratory infection, gastrointestinal symptoms, and headache) and rarely serious (gastrointestinal perforations, hemorrhage, and thrombolysis) adverse effects.

### 7.3. Cetuximab (Erbitux)

Cetuximab (Merck, ImClone, Briston-Myers-Squibb) is a recombinant, human-IgG1/mouse chimeric monoclonal antibody which blocks phosphorylation and activation of receptor-associated kinases by binding to the receptor. Erbitux is single-used or used in combination with other therapies to treat CRC. The US FDA used three separate clinical trials as a base to approve Erbitux for treatment of EGFR-expressing, recurrent metastatic CRC in patients who are intolerant to irinotecan-based chemotherapy in 2004. In 2007, the US FDA expanded labeling and granted regular approval for single-agent cetuximab for the treatment of patients with EGFR-expressing metastatic CRC after failure of both irinotecan- and oxaliplatin-based chemotherapy regimens (http://www.cancer.gov/). 

Known side-effects are rash, asthenia/malaise, diarrhea, nausea, abdominal pain, vomiting, fever, and infusion reaction [159–162].

### 7.4. Panitumumab (Vectibix)

Panitumumab (Amgen), a recombinant, human IgG2 kappa monoclonal antibody, binds specifically to the extracellular domain of EGFR and thereby prevents its activation and downstream signaling sequeale [163–166]. In 2006, panitumumab was approved by the US FDA for treatment of EGFR-expressing metastatic CRC with disease progression despite prior treatment; in 2008 by the EMEA for the treatment of refractory EGFR-expressing metastatic CRC in patients with nonmutated K-Ras. 

Known side-effects include dermatological toxicities, ocular toxicities, hypomagnesemia, fatigue, abdominal pain, nausea, diarrhea and constipation.

### 7.5. VEGF-Trap (ZALTRAP, Aflibercept), HuMV833, and Other Monoclonal Antibodies Targeting VEGF

VEGF-trap (Sanofi-Aventis and Regeneron) is a soluble decoy receptor protein consisting of a hybrid Fc construct in which domain 2 of VEGFR-1 is fused to domain 3 of the VEGFR-2 [167, 168]. VEGF-trap is known to have high affinity to all isoforms of VEGF-A. It caused vessel-regression of coopted vessels in a model of neuroblastoma [169]. Several clinical phase II/III trials testing the VEGF-trap in solid and hematologic malignancies including CRC, MM, pancreatic cancer, prostate cancer, NSCLC are ongoing (http://clinicaltrials.gov/). On April 26, 2011, Sanofi-Aventis and Regeneron reported about the positive phase III results with VEGF-trap in second-line mCRC. The VELOUR study evaluates ZALTRAP in combination with FOLFIRI chemotherapy *versus* FOLFORI plus placebo. Exact results are eagerly awaited for the second half of 2011. 

Similarly, HuMV833, a humanized monoclonal IgG antibody-binding VEGF-A isoforms (VEGF121 and VEGF165), demonstrated antitumor effects in a variety of human tumor xenograft models [170, 171]. 

Additionally, antibodies against VEGFR-1 or VEGFR-2 (IMC-18F1, IMC-1121B, ImClone) are under preclinical and clinical investigation. IMC-18F1 is a fully human, high affinity neutralizing antibody that specifically blocks VEGFR-1 activation, which has demonstrated preclinical activity in BC [172]. IMC-1121B (ramucirumab), a fully human monoclonal IgG1 antibody against the extracellular domain of VEGFR-2, is currently under evaluation in various entities including advanced liver, kidney, prostate, ovarian, colorectal, melanoma, BC, and NSCL cancer [173, 174].

### 7.6. Trastuzumab (Herceptin)

Trastuzumab (Genentech) is a recombinant humanized monoclonal antibody which binds to the extracellular domain of the HER2 receptor and inhibits the intracellular tyrosine kinase activity. In addition, it blocks cleavage of HER2 and thereby the production of p95, interferes with either homodimerization or heterodimerization of HER2 with itself or other HER receptors, and recruits Fc-competent immune effector cells and other components of antibody-dependent cell-mediated cell cytotoxicity (ADCC). In 1998, trastuzumab was FDA approved for treatment of patients with HER2-positive metastatic BC in combination with paclitaxel. In 2006, FDA approval of trastuzumab was expanded for the adjuvant setting in combination with chemotherapy regimens containing doxorubicin, cyclophosphamide, and paclitaxel. In January 2008, FDA approval was revised to include the use of trastuzumab also as a single agent in the adjuvant setting [175].

### 7.7. Small Molecule Inhibitors

Although Avastin is an effective medication and studies testing the VEGF-trap or VEGFR-targeting antibodies are promising, drug resistance always develops likely due to targeting a single tumorigenic pathway. Indeed extended blockade of VEGF alone results in tumor revascularization, dependent on other proangiogenic factors such as FGF [176]. Small-molecule inhibitors have the advantage of being orally available as well as more promiscuous in target inhibition and also less expensive [177, 178]. Based on these therapeutic advantages, many tyrosine kinase inhibitors (TKIs) have been developed and subjected to clinical trials. Indeed, the second-generation multi-targeted receptor kinase inhibitors (RTKIs) sorafenib, sunitinib, and pazopanib have now been approved for the treatment of advanced RCC and gastrointestinal stroma tumor (GIST), hepatocellular carcinoma (HCC). Moreover, preliminary data in other malignancies, most prominently including CRC and BC are promising.

#### 7.7.1. Sorafenib (Nexavar)

Sorafenib (Bayer HealthCare Pharmaceuticals and Onyx Pharmaceuticals) [179, 180] is a RTK inhibitor which targets VEGFR2, VEGFR-3, Raf, PDGFR*β*, Flt3, and c-Kit. It was approved for the treatment of advanced RCC in 2005 and for the treatment of unresectable HCC in 2007. Advanced clinical studies in NSCLC and melanoma are ongoing.

#### 7.7.2. Sunitinib (Sutent)

Sunitinib (Pfizer) is another multitargeted TKI which targets VEGFR2, PDGFR*α*/*β*, c-Kit, Flt3, RET [181–184]. Based on a phase III clinical trial, in which sunitinib demonstrated improvements in progression-free survival when compared to IFN*α*, it was approved for first-line and second-line therapy of metastatic RCC [185, 186]. In addition, sunitinib was also approved for treatment of GIST in 2006 [187]. Advanced clinical studies are ongoing in breast, colorectal, and lung cancer. Both sorafenib and sunitinib alone or in combination therapy are under clinical evaluation in MM.

#### 7.7.3. Temsirolimus (Torisel) and Everolimus (Afinitor)

Temsirolimus (Wyeth Pharmaceuticals), a derivative of rapamycin, is a specific inhibitor of the mammalian target of rapamycin (mTOR). mTOR pathway has an important role in regulating the synthesis of HIF and proteins that control cell proliferation, such as c-myc and cyclin D1. Therefore, inhibiting mTOR in RCC downregulates HIF activity and stops the production of cell-cycle regulators [188, 189]. In 2007, Temsirolimus was approved for the treatment of advanced RCC. As compared with IFN*α*, temsirolimus improved overall survival among patients with mRCC and poor prognosis [190]. 

Everolimus (Novartis), another rapamycin analogue was approved for treatment of patients with mRCC whose disease had progressed despite prior treatment with sunitinib, sorafenib, or both in 2009 [191, 192]. 

Clinical studies which evaluate the activity of temsirolimus and everolimus in other tumor entities including BC, gastric cancer, HCC, MM, and lymphoma are ongoing.

#### 7.7.4. Pazopanib (Votrient)

Pazopanib (GlaxoSmithKline) is a novel orally available, small-molecule tyrosine kinase inhibitor of VEGF-receptor-1, -2, -3 with IC50's of 10, 30, and 47 nM, respectively. In 2010, pazopanib was approved as the third TKI and the last among the six treatments for mRCC (sorafenib, sunitinib, temsirolimus, everolimus, bevacizumab) approved by the FDA during the last 5 years. The basis for this approval was a randomized, double-blind, placebo-controlled phase III study evaluating the efficacy and safety of pazopanib in 435 patients with locally advanced and/or mRCC. The median PFS for the pazopanib was 9.2 months compared with 4.2 months for the placebo in overall population (*P* < 0.001) [193]. Moreover, the combination of pazopanib with lapatinib was effective in patients with BC, and preclinical data in MM were promising [194]. Clinical studies which evaluate the activity of pazopanib in other tumor entities are ongoing.

#### 7.7.5. Axitinib

Axitinib (Pfizer) is an oral, potent, and selective inhibitor of VEGFR 1–3, PDGFR*β*, and c-KIT. Promising data from a clinical phase I study [195] prompted the clinical evaluation of Axitinib in a variety of malignancies. Excitingly, clinical activity has now been demonstrated in sorafenib-refractory metastatic RCC [196] and patients with advanced NSCLC [197]. Moreover, a clinical phase III trial in patients with unresectable, locally advanced, or metastatic pancreatic cancer treated with gemcitabine plus axitinib is now ongoing to verify a small gain in overall survival observed in a clinical phase II trial [198]. Clinical trials in mCRC showed no benefit of axitinib in first- and second-line combination therapies with oxaliplatin-containing chemotherapies in comparison to bevacizumab [199].

Additional clinical studies are ongoing in GIST, lung cancer, thyroid cancer, and breast cancer. Dose-limiting toxicities primarily seen at higher dose levels included hypertension, hemoptysis, and stomatitis. The observed hypertension was manageable with medication. Stomatitis was generally tolerable and managed by dose reduction or drug holidays.

#### 7.7.6. Lapatinib (Tyverb)

Lapatinib (GlaxoSmithKline) is another orally available TKI inhibiting both EGFR and HER2 receptors [200–202]. It was FDA approved in 2007 for combination therapy for triple-positive BC patients already treated with capecitabine or which have progressed on trastuzumab. In 2010, lapatinib additionally received accelerated approval as front-line therapy in this patient cohort.

Side effects of Tyverb include diarrhea, palmar-plantar erythrodysesthesia, nausea, rash, vomiting, muscular inflammation, stomatitis, pain in extremities, dyspnea, and fatigue [203–206].

## 8. Discussion

Recent studies delineate a key role for the tumor microenvironment in tumorigenesis. Investigating the complex functional interrelation between the cellular and noncellular compartments of the tumor microenvironment has already led to the identification of new therapeutic targets. One pivotal compartment within the microenvironment is the vascular niche. Indeed, 40 years after Dr. Folkman's seminal postulation in 1971 that angiogenesis is required for tumor growth and progression and may therefore represent a new target for cancer therapy [18], it is well established that angiogenesis plays an important role in solid as well as in hematologic malignancies. Tumor angiogenesis is now recognized to be a hallmark of cancer, initiated by enhanced tumor/tumor-stroma cell-specific production of proangiogenic molecules, and/or suppression of antiangiogenic factors (angiogenic switch) as well as via tumor-associated hypoxia. The introduction of antiangiogenic agents into clinical practice was a milestone event in cancer therapy during the last decade.

VEGF, EGF, and PDGF represent key factors in tumor angiogenesis. Blocking BM angiogenesis in MM with thalidomide; and VEGF with the first-in-class antiangiogenic drug bevacizumab; or EGFR with cetuximab in CRC have become established anticancer strategies. Following the introduction of bevacizumab, efforts focused on the identification of compounds targeting VEGF signaling sequelae that can be given orally. Several second-generation orally available small-molecule antiangiogenic drugs have now been identified including sunitinib, pazopanib, and sorafenib and have recently been approved for treatment of cancers including CRC, BC, RCC, and MM. However the optimal use of antiangiogenics is tumor- and stage-dependent. Moreover, although antiangiogenic antibodies as well as small molecules targeting VEGF and EGF signaling pathways significantly prolong overall survival of cancer patients, resistance always develops and disease relapse is inevitable. Recent molecular mechanistic studies may explain the disappointing results of previous clinical studies using VEGF inhibitors *alone* either in early or *refractory/progressive* disease. Modest, though significant, survival benefits were observed in patients with *advanced* tumors treated with bevacizumab and other antiangiogenics *even when combined* with conventional chemotherapies. Further studies are needed to increase our understanding of tumor angiogenesis and of how resistance against antiangiogenic agents develops. Potential mechanisms of evasive resistance include the redundancy of proangiogenic signals in later disease stages; recruitment of vascular progenitor cells and proangiogenic monocytes from the bone marrow, increased and tight pericyte coverage, or increased capabilities for invasion and metastasis; preexisting inflammatory cell-mediated vascular protection; hypovascularity; invasive and metastatic cooption of normal vessels; and mutational alteration of genes within endothelial cells [207]. Therapeutic benefits may be achieved by initiating treatment with VEGF-inhibitors early: by using antiangiogenic cocktails, which not only target VEGF both in patients with early and late-stage disease, as well as metronomic therapy [208].

Novel approaches to improve antiangiogenic therapy include strategies to target the angiopoietin-TIE system, Hif-1, endothelial-specific integrin/survival signaling (e.g., by cilengitide) as well as the use of vascular-disrupting agents (VDAs), which selectively disrupt already existing tumor vessels by targeting dysmorphic endothelial cells. Given the benefits of combination therapy, it is also crucial to optimize existing or identify new treatment regimens in order to reduce drug-associated toxic side effects.

In summary, antiangiogenic compounds like thalidomide, bevacizumab, sorafenib, sunitinib, and pazopanib, temsirolimus and everolimus have already demonstrated activity in a variety of cancers most prominently including BC, CRC, RCC, and MM. However, with the increase of our knowledge of the complexity of molecular mechanisms contributing to tumor angiogenesis in general, and MM BM angiogenesis in particular, we aim to identify additional therapeutic targets, to further optimize treatment regimens; and to reduce mechanisms leading to antiangiogenic drug-resistance in order to further improve patient outcome and reduce drug toxicity.

## Figures and Tables

**Table 1:** Tumor microenvironment and its compartments.

	Tumor entities	
Microenvironment	Epithelial solid tumors	Hematological tumors
	For example, Breast Cancer	For example, multiple myeloma

Extracellular matrix (ECM)	fibronectin, laminin, collagen, proteoglycans, thrombospondin, fibrinogen, elastin, fibrin, tenascin, tetranectin	fibronectin, laminin, collagen, proteoglycans, glycosaminoglycans

Cellular	Hematopoietic: TAM, T and B lymphocytes, neutrophils, NK cells, mesenchymal stem cells	Hematopoietic: HSCs, BM-derived CEPs, hematopoietic and mesenchymal progenitor and precursor cells, NK cells, NKT cells, macrophages, T and B lymphocytes, DCs, monocytes, platelets, megakaryocytes, erythrocytes
	nonhematopoietic: CAFS, myoepithelial cells, ECs, pericytes	nonhematopoietic: fibroblasts/BMSCs, chondrocytes, OCs, OBs, ECs

Liquid	Hormones: estrogen, progesterone	
	cytokines and growth factors: VEGF, HGF/SF, bFGF, PDGF*α*/*β*, TGF*α*/*β*, IL-1, IL-6, TNF*α*, GM-CSF, CSF-1, IGF-1/2, EGF, SDF-1	Cytokines and growth factors: VEGF, IGFs, TNF*α*, CD40, IL-1, IL-6, IL-10, IL-11, IL-15, IL-21, HGF, bFGF, SDF-1, TGF*β*, LIF, OSM, MIP-1*α*, Wnts
	proteases: cathepsin B and D, elastase, uPA, plasmin, MMPs (e.g., MMP-1, -2, -3, -9)	proteases: uPA, plasmin, MMPs (e.g., MMP-2, -9)

TAM: tumor-associated macrophage; NK: nature killer; CAFS: cancer-associated fibroblasts; EC: endothelial cell; HSC: hematopoietic stem cells; CEP: circulating endothelial precursor; NKT: nature killer T; DC: dendritic cell; BMSC: bone marrow stromal cell; OC: osteoclast; OB: osteoblast; VEGF: vascular endothelial growth factor; HGF/SF: hepatocyte growth factor/scatter factor; bFGF: basic Fibroblast Growth Factors; PDGF: platelet-derived growth factor; TGF: transforming growth factor; TNF: tumor necrosis factor; IL: interleukin; GM-CSF: granulocyte macrophage colony stimulating factor; CSF: colony stimulating factor; EGF: epidermal growth factor; SDF: stromal cell-derived factor; uPA: urokinase plasminogen activator; MMP: matrix metalloproteinase; IGF: Insulin-like growth factor; LIF: leukaemia inhibitory factor; OSM: oncostatin M; MIP-1*α*: macrophage inflammatory protein 1*α*.

References for breast cancer: [8–11].

References for multiple myeloma: [12].

**Table 2:** Summary of drugs, their revealed targets and indications in clinical trails. Drugs without a single treatment trial are marked with a “*”.

Drug (brand name, company)	Target	Approved	Clinical trials with single treatment	Indication
Bevacizumab (Avastin, Genentech/Roche)	Monoclonal antibody against VEGFA	mCRC, mRCC, NSCLC, metastatic HER2-negative breast cancer, glioblastoma	Phase I, II	Multiple solid tumors (e.g., RCC, BC, pancreatic, prostate, ovarian, brain cancers) and hematologic malignancies (e.g., MM)

Sunitinib, SU11248 (Sutent, Pfizer)	TKI of VEGFR 1–3, PDGFR*α*/*β*, c-Kit, Flt3, RET, CSF-1R	mRCC, GIST	Phase I	Multiple solid tumors (e.g., RCC, BC, melanoma, lung)

Pazopanib (Votrient, GlaxoSmithKline)	TKI of VEGFR 1–3, PDGFR*α*/*β*, c-kit tyrosine kinases	mRCC	Phase I, II	Multiple solid tumors (e.g., BC, RCC, ovarian, lung) and others (e.g., lymphoma)

Sorafenib, BAY43-9006 (Nexavar, Bayer)	TKI of Multiple cell surface kinases (VEGFR 1–3, RET, PDGFR*β*, Flt-3, c-Kit, CSF-1) and intracellular kinases (CRAF, BRAF, mutant BRAF)	mRCC, unresectable hepatocellular carcinoma	Phase I, II	Multiple solid tumors (e.g., RCC, BC, melanoma, lung cancers) and hematologic malignancies (e.g., MM)

Vandetanib, ZD6474 (Zactima, AstraZeneca)	TKI of VEGFR, EGFR and RET	Metastatic medullary thyroid cancer	Phase I, II	NSCLC, RCC, glioblastoma

Bortezomib, PS-341 (Velcade, Millennium Pharmaceuticals)	26S proteasome inhibitor	MM, relapsed mantle cell lymphoma	Phase I, II	MM, lymphoma, leukemia and multiple solid tumors (e.g., RCC, BC, lung, prostate)

Temsirolimus (Torisel, Wyeth)	mTOR inhibitor	mRCC	Phase I, II	Multiple solid tumors (e.g., RCC, BC, melanoma, prostate, liver cancers) and hematologic malignancies (e.g., lymphoma)

Everolimus, RAD001 (Afinitor, Novartis)	mTOR inhibitor	Advanced renal cell carcinoma	Phase I, II	Multiple solid tumors (e.g., BC, pancreatic, gastric cancers) and lymphoma

Thalidomide (Thalomid, Celgene)	Angiogenesis inhibitor, multiple	MM	*	MM

Lenalidomide, CC-5013 (Revlimid, Celgene)	Angiogenesis inhibitor, Thalidomide derivative	MM	Phase I, II	MM, lymphoma, chronic lymphocytic leukemia, and multiple solid tumors (e.g., CRC, ovarian)

Pomalidomide, CC-4047 (Actimid, Celgene)	Angiogenesis inhibitor, Thalidomide derivative	No yet approved	Phase I	MM, Lymphoma

Aflibercept, VEGF-trap (ZALTRAP, Sanofi-Aventis and Regeneron)	Decoy receptor for all VEGF-A isoforms	No yet approved	*	mCRC, RCC, Ovarian, NSCLC, prostate cancers, lymphoma, leukemia

Axitinib, AG-013736 (Pfizer)	TKI of VEGFR 1–3, PDGFR*β*, c-KIT and CSF-1	No yet approved	Phase I	mRCC, BC, NSCLC, metastatic pancreatic cancer, GIST, lung cancer, thyroid cancer

Icrucumab, IMC-18F1 (ImClone)	Monoclonal antibody against VEGFR-1	No yet approved	Phase I	Advanced solid tumors, (e.g., CRC, BC, carcinoma of urinary tract)

Ramucirumab, IMC-1121b (ImClone)	Monoclonal antibody against VEGFR-2	No yet approved	*	CRC, BC, mRCC, Advanced liver, gastric, prostate, ovarian, and NSCL cancers, melanoma

Vatalanib, PTK787 (Novartis)	TKI of VEGFR 1–3, PDGFR*α*/*β*, and c-KIT	Not yet approved	*	Multiple solid tumors (e.g., CRC, glioblastoma, NSCLCs) and hematologic malignancies (e.g., leukemia)

Enzastaurin, LY317615.HCl (Eli Lilly)	PKC inhibitor	Not yet approved	*	BC, mCRC, Brain tumor, advanced NSCL, glioblastoma, lymphoma

Cediranib, AZD2171 (Recentin, AstraZeneca)	TKI of VEGFR 1–3	Not yet approved	Phase I	RCC, CRC, BC, ovarian, prostate cancer, lung, brain, head and neck cancers, glioblastoma, melanoma

Vectibix, panitumumab (Amgen)	EGFR	mCRC	Phase I, II	mCRC, pancreatic, HNSCC, NSCLC, lung

Erbitux, cetuximab, (Imclone, Bristol-Myers Squibb)	EGFR	mCRC	Phase I, II	mCRC, HSNCC, brain, MM, lung, pancreatic, liver

Trastuzumab, herceptin (Genentech)	HER2 receptor	Gastric cancer, HER2 positive BC	Phase I, II	BC, gastric

Tykerb, lapatinib (GlaxoSmithKline)	EGFR and HER2 receptor	BC	Phase I, II	BC, CRC, lung, HNSCC, pancreatic, melanoma

Tamoxifen, Novadex, Istubal, Valodex (AstraZeneca)	Estrogen receptor	BC	Phase I, II	BC, bladder, melanoma, prostate

TKI: tyrosine kinase inhibitors; mCRC: metastatic colorectal cancer; NSCLC: nonsmall cell lung cancer; mRCC: metastatic renal cell carcinoma; GIST: gastrointestinal stroma tumor after progression; MM: multiple myeloma; BC: breast cancer; HNSCC: head and neck sequmous cell carcinoma.
